# A Seamless Gene Deletion Method and Its Application for Regulation of Higher Alcohols and Ester in Baijiu* Saccharomyces cerevisiae*

**DOI:** 10.1155/2019/6723849

**Published:** 2019-05-09

**Authors:** Ping Li, Junling Ge, Yingying Gao, Jianhui Wang, Cuiying Zhang, Dongguang Xiao

**Affiliations:** ^1^State Key Laboratory of Food Nutrition and Safety, Key Laboratory of Industrial Fermentation Microbiology, Ministry of Education, Tianjin Industrial Microbiology Key Laboratory, College of Biotechnology, Tianjin University of Science and Technology, Tianjin 300457, China; ^2^Key Laboratory of Wuliangye-flavor Liquor Solid-state Fermentation, China National Light Industry, China

## Abstract

The security of engineering* Saccharomyces cerevisiae* is becoming more focused on industrial production in consideration of the public concern regarding genetically modified organisms. In this work, a rapid and highly efficient system for seamless gene deletion in* S. cerevisiae *was developed through two-step integration protocol combined with endonuclease I-SCEI expression. The factors affecting the frequency of the second homologous recombination were optimized, and studies indicated that the mutant strains with 500 bp direct repeats and that have been incubating in galactose (0.5 g/100 mL) medium at 30°C and 180 r/min for 24 h permit high frequency (6.86 × 10^−4^) of the second homologous recombination. Furthermore, DNA sequence assays showed only self-DNA in native location without any foreign genes after deletion using this method. The seamless gene deletion method was applied to the construction of the engineering strains with* BAT2 *(encoding aminotransferase) deletion and* ATF1 *(alcohol acetyltransferases) overexpression. The mutants exhibited significant effects on higher alcohol reduction and ester improvement after Baijiu fermentation. The engineered strains can be used in industrial production in security, thereby meeting the requirements of modern science and technology.

## 1. Introduction

*Saccharomyces cerevisiae* is widely used for industrial production as an efficient and important fermentation microorganism because of its advantageous qualities, such as high-ethanol productivity, tolerance to process hardiness, and tolerance to fermentation byproducts; therefore, it is preferred for crop ethanol production [1–3]. Meanwhile, with the public's increasing concern about genetically modified (GM) organisms, the security of genetically engineered strains has attracted an increasing amount of attention.

Baijiu (Chinese liquor) presents peculiar sensory characteristics and has health-promoting effects.* S. cerevisiae* is the dominant microorganism in Baijiu fermentation and is closely related to the quality of Baijiu.* S. cerevisiae *is not only responsible for the efficient production of ethanol, it can also be manipulated to produce other heterologous flavor substances during alcoholic fermentation. Acetate esters and higher alcohols are significant parameters in the determination of the beverage's quality and flavor profiles [4–6]. Higher alcohols can lead to fusel oil taste and cause potential damage to human health at high levels [7]. Acetate esters, such as ethyl acetate, isoamyl acetate, and isobutyl acetate, are among the flavor-active esters that can elicit the desired fruity aroma in alcoholic drinks [8].

Systematic sequencing of* S. cerevisiae *genome has revealed a profusion of Open Reading Frames, and a large majority of genes need to be mediated to determine its phenotypic effects [9, 10]. To date, several genome editing protocols have been widely employed and developed for* S. cerevisiae *[11–16]. Cre/loxP recombination is used as a site-specific recombinase technology for controlling gene expression; it relies on the integration of 34-bp* loxP* sequences directly upstream and downstream of the genomic target by one double homologous or two single homologous recombination events. The cassette can precisely be removed with the induction of Cre recombinase and used again for the succeeding deletion step. However, a scar is left in the chromosome in the form of* loxP* site after each deletion step, thereby affecting the efficiency of the next gene deletion. Recent research has demonstrated that CRISPR-associated (Cas) systems can serve as the basis of a simple and highly efficient method for manipulating the genome in bacteria, yeast, and human cells [17–21]. The CRISPR-associated enzyme Cas9 is an RNA-guided endonuclease that uses RNA:DNA base-pairing to target foreign DNA [22–24]. However, the key limitation of this system is off-target effects. Single guide RNA (SgRNA), which guides the CRISPR/Cas9 to interrogate the genome and matches the targeted DNA fragment, can tolerate certain mismatches to the DNA targets, thereby promoting undesired off-target mutagenesis [25–27]. To solve these problems, an improved and efficient system should be developed for genome editing in* S. cerevisiae*.

Repair of chromosomal double-strand breaks (DSBs) can be accomplished through homologous recombination in their immediate vicinity on the chromosome in a variety of organisms [28, 29]. Galactose-inducible I-SCE1 endonuclease can be used to incorporate an inducible DSB feature [30]. In the current study, we developed a rapid and highly efficient system through two-step integration protocol combined with endonuclease I-SCEI expression to accelerate the pace and efficiency of progress of seamless gene deletion in Baijiu* S. cerevisiae*. Furthermore, the factors affecting the frequency of the second homologous recombination efficiency, including the length of direct repeat (DR) sequences, induction time, and galactose concentration, were screened and optimized. Using the toolkit, the* S. cerevisiae* with* BAT2 *(encoding aminotransferase) deletion and* ATF1 *(encoding alcohol acetyltransferases) overexpression were engineered to reduce the higher alcohol concentration and improve the acetate ester production in Baijiu. This study could serve as a good reference for genome editing and future optimization of yeast strains in alcoholic beverages.

## 2. Materials and Methods

### 2.1. Strains, Culture Conditions, and Media

Strains used in this work are shown in Table 1.* Escherichia coli *was used to construct plasmids.* S. cerevisiae *strain *α*5 was used as the parent strain; this was cultured in yeast extract peptone dextrose (YEPD) (1% yeast extract, 2% peptone, and 2% glucose) for general culturing and in galactose medium (1% yeast extract, 2% peptone, 100 mg/L adenine hemisulfate, and 1% galactose) for galactose induction. Selection and counter-selection were performed in the medium TPGly + AF (1% yeast extract, 2% peptone, 5% glycerol, 200 mg/mL methotrexate, 5 mg/mL sulfanilamide, 5 mg/mL thymidine, and 50 mg/mL hypoxanthine) and the medium SC + FUdR [0.17% YNB, 0.5% (NH_4_)_2_SO_4_, 2% glucose, 50 *μ*g/mL 5-fluorodeoxyuridine (FudR)], respectively.

### 2.2. Plasmid Construction

The primers designed in this study are listed in Table 2. The plasmid pUC19-UD was constructed as follows. pUC19 was used as backbone. Upstream* BAT2-U(1500)* and downstream* BAT2-D(1500)* were amplified from yeast strain *α*5 with primers BAT2-A(KpnI)-F/BAT2-A-R and BAT2-B-F/BAT2-B(SphI)-R, respectively. Fusion polymerase chain reaction (PCR) with a mixture of upstream and downstream sequences was carried out. Then, the PCR product was* Kpn*I-*Sph*I double-digested and inserted in the same* Kpn*I-*Sph*I digested plasmid pUC19, thereby creating the plasmid pUC19-UD.

**Figure 1:**
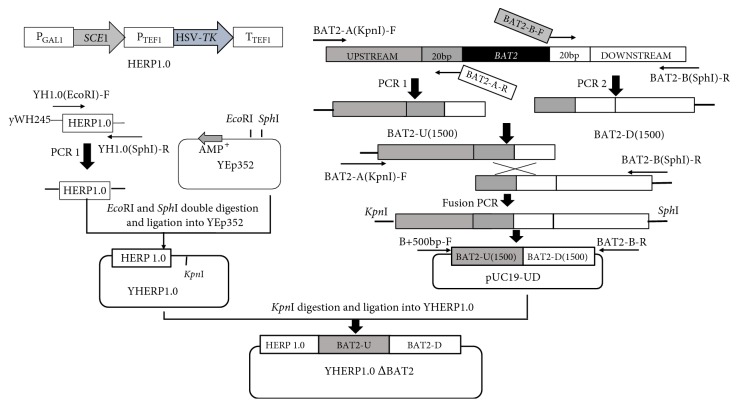
Construction of plasmid YHERP1.0ΔBAT2.

The plasmids YHERP1.0ΔBAT2 and YHERP1.0ΔBAT2::ATF1 were constructed as follows. First, the HERP1.0 (*P*_*GAL1*_*-I-SCEI-P*_*TEF1*_*-HSV-TK-T*_*TEF1*_) was amplified using the primers YH1.0(EcoRI)-F/YH1.0(SphI)-R from the yeast yWH245 gifted by William G [30]. The cassette was inserted into* EcoR*I-*Sph*I-digested plasmid Yep352, creating the plasmid YHERP1.0. Second, the fragment of* BAT2-UD *(1183 bp) amplified from pUC19-UD with the primers B+500 bp-F/BAT2-B-R and the fragment* BA-P*_*TEF1*_*-ATF1-T*_*PGK1*_*-BB *(3600 bp) amplified from pUC-BBTAP preserved in this laboratory by the primers YH1.0ΔB::A-F/YH1.0ΔB::A-R were cloned into the plasmid YHERP1.0 digested with* Kpn*I, obtaining the plasmids YHERP1.0ΔBAT2 (Figure 1) and YHERP1.0ΔBAT2::ATF1.

### 2.3. First Step of Integration of HERP1.0 Cassette

Primers BAT2-A-F and BAT2-A-R(F) [containing the restriction site for I-SCEI (S) and 27 bp homologous sequence (F) of the HERP1.0] were used to amplify the fragment* BAT2-A(RSF)* from the yeast *α*5 chromosome. Then, the fragment* RS-HERP1.0-BAT2-UD* was amplified with the primer BAT2-B-F(R) [containing the restriction site for I-SCEI (S) and homologous sequence (R) of BAT2-A(SF)] and BAT2-B-R from plasmid YHERP1.0ΔBAT2. In addition,* RS-HERP1.0-BA-P*_*TEF1*_*-ATF1-T*_*PGK1*_*-BB* fragment was amplified from the plasmid YHERP1.0ΔBAT2::ATF1 by using the primers BAT2-B-F(R) and YH1.0ΔB::A-R. The primers used in first step of integration of HERP1.0 cassette are shown in Table 2.

The fragments* BAT2-A(RSF)* and* RS-HERP1.0-BAT2-UD* were transformed into the yeast strain *α*5 by lithium acetate method and spread onto TPGly + AF plates, obtaining a mutant yeast H*α*5 + H (*BAT2* deletion) with HERP1.0 integrant. Using the same method, fragments* BAT2-A(RSF)* and* RS-HERP1.0-BA-P*_*TEF1*_*-ATF1-T*_*PGK1*_*-BB* were transformed into the yeast strain *α*5 to construct mutant H*α*5::ATF1+H (*BAT2* deletion and* ATF1 *overexpression) with HERP1.0 integrant.

### 2.4. Pop-Out of HERP1.0 Cassette through Second Step of Induction in Galactose

**Figure 2:**
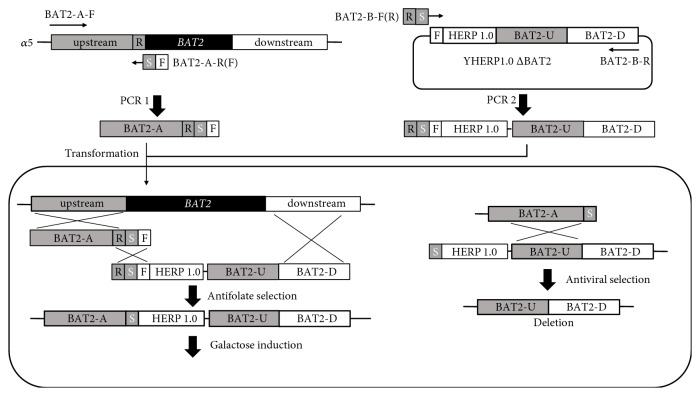
Schematic illustration of seamless deletion of* BAT2* with two-step homologous recombination in* Saccharomyces cerevisiae*.

H*α*5 + H cells were incubated and induced in galactose medium at 30°C at 180 rpm. The I-SCE1 endonuclease was expressed at* I-SCE1* site, and DSB was generated and repaired through the second homologous recombination. The yeast solution after dilution was spread onto SC + FUdR. The resulting strain H*α*5 popping out the HERP1.0 integrant was constructed (Figure 2). The mutant H*α*5::ATF1 (*BAT2* deletion and ATF1 overexpression) was constructed using the same processing step.

### 2.5. Optimization of Factors Affecting Frequency of the Second Homologous Recombination

Different lengths of DRs (50, 150, 300, 500, 700, and 1000 bp) were used to construct the plasmids YHERP1.0(50), YHERP1.0(150), YHERP1.0(300), YHERP1.0(500), YHERP1.0(700), and YHERP1.0(1000). The fragments* RS-HERP1.0-BAT2-U *(50, 150, 300, 500, 700, and 1000) D amplified from the plasmids YHERP1.0 (50, 150, 300, 500, 700, and 1000) were transformed into the yeast strain *α*5 with the fragment BAT2-A-SF, thereby yielding the mutants H*α*5 + H (50, 150, 300, 500, 700, and 1000) that carry HERP1.0 integrants with different DR lengths. The mutants were induced in 0.1 g/100 mL galactose for approximately 24 h and replica-plated onto TPGly + AF and YEPD plates.

Chromosomal DSBs were generated through I-SCEI endonuclease induction. To confirm the effect of galactose-inducible time on the frequency of the second homologous recombination efficiency, the mutants carrying the HERP1.0 integrant with 500 bp DRs were induced in 0.1 g/100 mL galactose medium for 12, 24, 36, and 48 h and replica-plated onto TPGly + AF and YEPD plates. The counts of the mutants were measured in both plates.

The media with different galactose concentrations (0.1, 0.5, 1, 2, 3, 4, and 5 g/100 mL) were used to induce I-SCEI endonuclease expression. The mutants carrying HERP1.0 integrant with 500 bp DRs were induced in the galactose media for 24 h and replica-plated onto TPGly + AF and YEPD plates. The growth and characteristics of colonies cultured in TPGly + AF and YEPD plates were measured.

### 2.6. Fermentation Experiment

Corn hydrolysate medium was used in Baijiu fermentation of the parental strain and mutants. The details of the experiment procedures, including preparation of corn hydrolysate and control of the fermentation process, were based on those used in a previous study [31]. All fermentations were performed in triplicate.

### 2.7. Analytical Methods

CO_2_ weight loss during fermentation was determined using an analytical balance. Residual sugar, ethanol production, total acids, and total ester were detected after the fermentation was terminated using the standard method according to the International Organization of Vine and Wine [32]. The production of volatile flavor compounds including higher alcohols and esters was determined using Agilent 7890C GC through the method reported by Ma et al. [33].

### 2.8. Statistical Analysis

Data were represented as the mean ± standard errors. The differences between the transformants and the parental strain were confirmed by Student's t-test. Statistical significance was considered at* P* < 0.05.

## 3. Results

### 3.1. Construction of the Seamless Gene Deletion Method

In the current study, a seamless gene deletion system was constructed using the* BAT2* gene as a target gene. In HERP1.0 cassette,* TkMX *was fused to the* I-SCE1* gene driven by the galactose-inducible promoter of* GAL1 *by gap repair cloning in the strain. Fragments* BAT2-A(RSF)* and* RS-HERP1.0-BAT2-UD* were transformed into the *α*5 chromosome to replace* BAT2* gene through the first homologous recombination. After galactose induction, DSBs by I-SCE1 endonuclease induction facilitated the second homologous recombination, and the HERP1.0 cassette was popped out. The process of deletion strategy is illustrated in Figures 1 and 2.

**Figure 3:**
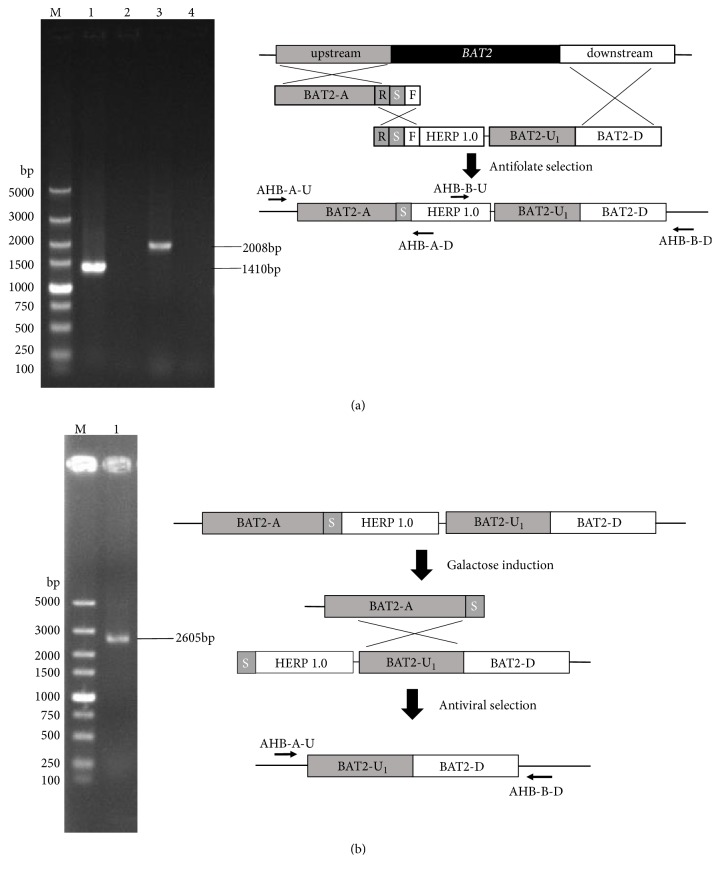
*Colony PCR of integrants*. (a) PCR verification of first step of integration of HERP1.0 cassette. (b) PCR verification of pop-out of HERP1.0 cassette.

After transformation of fragments* BAT2-A(RSF)* and* RS-HERP1.0-BAT2-UD*, the correct mutant H*α*5+H (*BAT2* deletion) with HERP1.0 integrant was selected on TPGly + AF plates. PCR amplification with specific primers (AHB-A-U and AHB-A-D, AHB-B-U, and AHB-B-D) from genomic DNA of desired mutant strains produced a 1410 bp band (Figure 3(a), lane 2) and a 2008 bp band. Moreover, no amplification (Figure 3(a), lane 1) was observed from the parental strain *α*5. The fragments* BAT2-A(RSF)* and* RS-HERP1.0-BAT2-UD* were integrated at the target site of genome in* S. cerevisiaeα*5.

**Figure 4:**
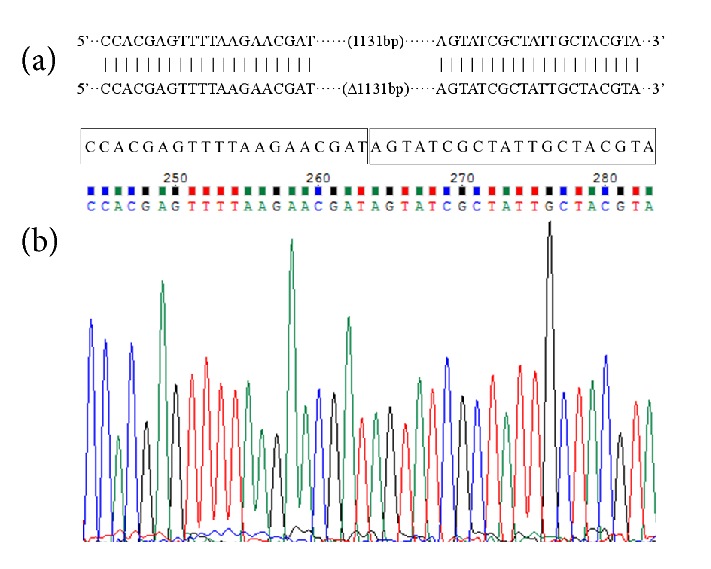
*Sequence alignment and sequencing results of the adjacent region of BAT2 after pop-out of HERP1.0 cassette*. (a) Comparison of the sequences before (above) and after (below) deletion. (b) Sequencing results of deletion.

H*α*5 + H cells were cultivated and induced in galactose medium and then replica-plated onto SC + FUdR plates. Only strains in which the HERP1.0 integrant was popped out can grow on SC + FUdR plates. The deletion strains were further confirmed via PCR by using the primer pairs of AHB-A-U and AHB-B-D. A 2605 bp band (Figure 3(b), lane 1) containing no HERP1.0 region was obtained, demonstrating that the strain H*α*5 with* BAT2* seamless deletion was constructed successfully. In addition, the sequence alignment was detected, and the sequencing results illustrated in Figure 3 are consistent with the S288C genomic sequence from the* Saccharomyces* Genome Database (SGD, accession number 9169867). The 1131 bp sequence containing the gene* BAT2* region of genome was completely deleted, and no foreign DNA sequence was retained in the* S. cerevisiae* chromosome (Figure 4, part of results).

### 3.2. Effect of the Factors on Frequency of the Second Homologous Recombination Efficiency

**Figure 5:**
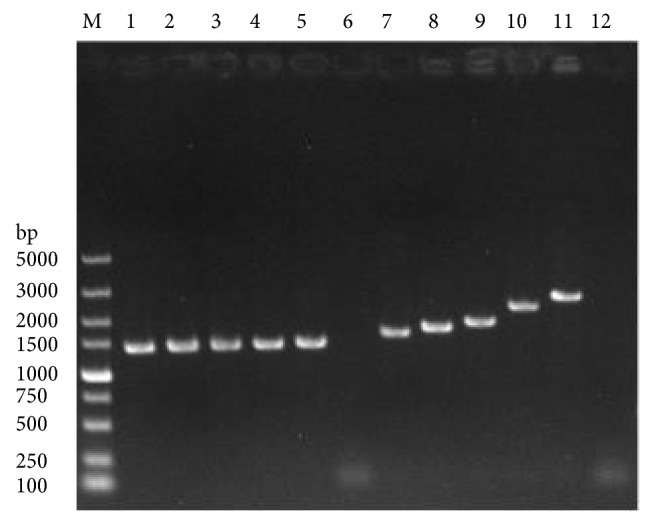
*PCR verification of mutant strains Hα5+H (50, 150, 300, 700, 1000)*.* M* DL5000 DNA marker;* lanes 1*-*lanes 6* PCR verification results from mutant strains H*α*5+H (50), H*α*5+H (150), H*α*5+H (300), H*α*5+H (700), H*α*5+H (1000), parental strain, respectively, using primer pairs AHB-A-U and AHB-A-D;* lanes 7*-*lanes 12* PCR verification results from mutant strains H*α*5+H (50), H*α*5+H (150), H*α*5+H (300), H*α*5+H (700), H*α*5+H (1000), parental strain, respectively, using primer pairs AHB-B-U and AHB-B-D.

**Figure 6:**
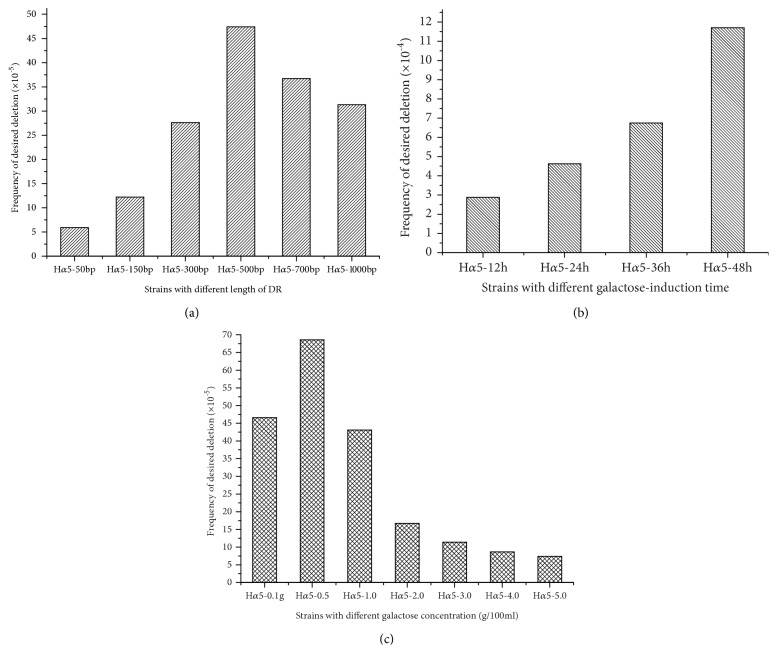
The effect of the length of DRs (a), induction time in galactose (b), and galactose concentration (c) on frequency of the second homologous recombination efficiency.

Different lengths of DRs (50, 150, 300, 500, 700, and 1000 bp) were used to detect the second homologous recombination efficiency. The first homologous recombination, in which mutants H*α*5 + H (50, 150, 300, 500, 700, and 1000) carrying HERP1.0 integrants with different lengths of DRs were constructed, was verified via PCR by using primer pairs of AHB-A-U and AHB-A-D and AHB-B-U and AHB-B-D (Figure 5). Regarding galactose induction, the second homologous recombination was performed, and the counts of yeast colonies were measured in TPGly + AF and YEPD plates. The result is shown in Figure 6(a). When the DR length was less than 500 bp, the second homologous recombination efficiency increased with increasing DR length but showed no obvious distinction when the DR length exceeded 700 bp. The desired deletion occurred at a frequency of approximately 4.74 × 10^−4^ at the optimal length (500 bp) of DRs.

Mutant strains carrying HERP1.0 integrant with 500 bp DRs were induced in galactose for 12, 24, 36, and 48 h to confirm the effect of galactose-inducible time on chromosomal DSBs. The count of the mutants was measured in TPGly + AF and YEPD plates.  Figure 6(b) shows that the second homologous recombination efficiency increased with increasing of galactose induction time. Meanwhile, long experimental time has a direct effect on experimental period. The induction time of 24 h was chosen as the optimal induction time, and the second homologous recombination efficiency was 4.62 × 10^−4^.

As the I-SCEI endonuclease expression was affected by galactose concentration, mutants carrying HERP1.0 integrant with 500 bp DRs were induced in media with different galactose concentrations. The colonies in TPGly + AF and YEPD plates were counted. The result in Figure 6(c) demonstrates that the galactose content had a significant effect on the second homologous recombination. At the galactose concentrations of 0.1 and 0.5 g/100 mL, the second homologous recombination efficiencies were 4.66 × 10^−4^ and 6.86 × 10^−4^, respectively, showing an increased tendency. However, when the content was in the range of 1 g/100 mL to 5 g/100 mL, the second homologous recombination efficiency decreased. The 0.5 g/100 mL content was chosen as the optimal condition to induce the I-SCEI endonuclease expression to generate chromosomal DSBs.

### 3.3. Regulation of* BAT2* Seamless Deletion and* ATF1* Overexpression on Higher Alcohols and Ester in Baijiu* S. *cerevisiae

Branched-chain alcohols are products from the degradation of corresponding branched-chain amino acids (BCAAs). BCAAs are converted to the corresponding *α*-keto acids through the initial transamination step, which is catalyzed by the aminotransferases (encoded by* BAT2*) [34]. Alcohol acetyltransferase encoded by gene* ATF1* (known as AATaseI or Atf1p) is a key enzyme in the synthesis of acetate ester, which is one of the major beneficial esters and responsible for the highly desired fruity aroma in Baijiu. Thus, fragment* P*_*TEF1*_*-ATF1-T*_*PGK1*_, in which the* ATF1* gene was overexpressed under the control of the* TEF1 *promoter, was inserted into the locus of* BAT2* gene to construct the strain H*α*5::ATF1 with* BAT2* gene deletion and* ATF1* gene overexpression.

**Figure 7:**
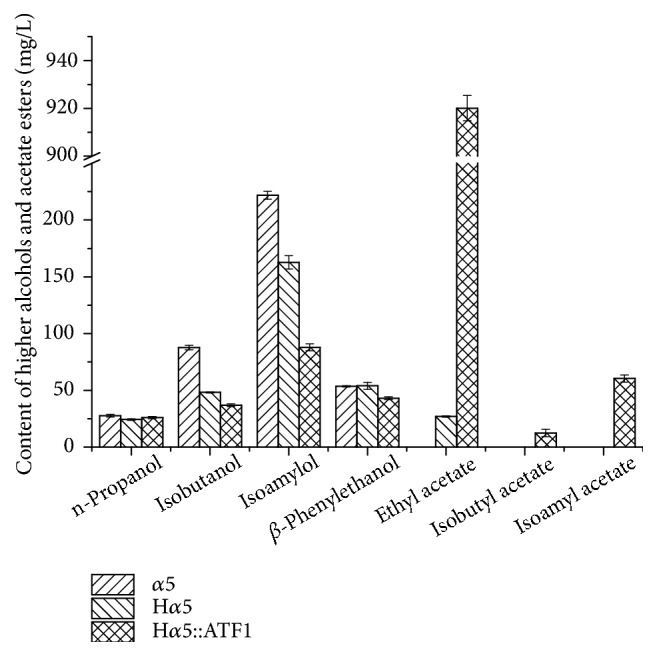
*Higher alcohols and esters production of the recombinant strains and parental strain in Baijiu*. Data are the average of three independent experiments. Error bars represent ± SD.

The mutant strains H*α*5 (*BAT2 *gene deletion) and H*α*5::ATF1 (*BAT2 *gene deletion and* ATF1* gene overexpression) were fermented in Baijiu in contrast with the parental strain. Moreover, the production of higher alcohols and esters were detected after fermentation to confirm the effect of the mutants H*α*5 and H*α*5::ATF1 on the regulation of higher alcohols and esters (Figure 7). No obvious distinctions were obtained in acetate esters and *β*-phenylethanol content of the parental strain and H*α*5 (*P *> 0.05). The production of n-propanol, isobutanol, and isoamylol by H*α*5 were 24.25, 48.26, and 162.65 mg/L, which decreased by 20.32%, 44.92%, and 26.64%, respectively, compared with those of the parental strain (30.44, 87.62, and 221.70 mg/L,* P *< 0.05). This result showed the significant effects on higher alcohol reduction. The concentration of ethyl acetate by H*α*5::ATF1 was 920.05 mg/L, which was 34.84-fold higher than that produced by parental strain *α*5 (25.68 mg/L,* P *< 0.05). Moreover, the content of isobutyl acetate and isoamyl acetate increased to 12.43 and 60.38 mg/L, respectively. The *β*-phenylethanol, isobutanol, and isoamylol contents produced by H*α*5::ATF1 were 21.15% (52.32 mg/L in the parental strain and 41.25 mg/L in H*α*5::ATF1,* P *< 0.05), 57.85% (87.62 mg/L in the parental strain and 36.93 mg/L in H*α*5::ATF1,* P *< 0.05) and 60.36% (221.70 mg/L in the parental strain and 87.86 mg/L in H*α*5::ATF1,* P *< 0.05) less than those produced by parental strain *α*5. Moreover, compared with mutant H*α*5, the isobutanol and isoamylol contents by H*α*5::ATF1 decreased by 23.48% (48.26 mg/L in H*α*5,* P *< 0.05) and 45.98% (162.65 mg/L in H*α*5,* P *< 0.05), respectively. This result demonstrated that the mutant strain H*α*5::ATF1 had significant effect not only on higher alcohol reduction but also on acetate ester improvement in* S. cerevisiae*.

### 3.4. Fermentation Properties of the Mutant Strains

**Figure 8:**
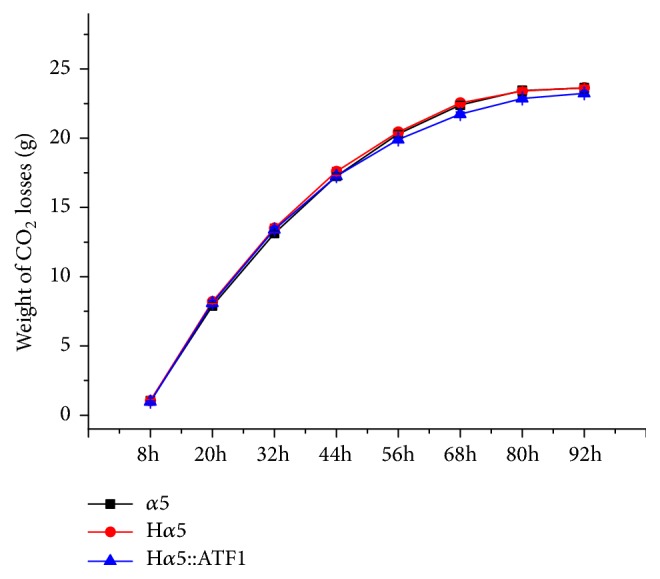
*Weight loss of mutant strains and parental strain during small-scale Baijiu fermentation*. Data are the average of three independent experiments. Error bars represent ± SD.

Stable and credible performance of the strains is remarkably important for Baijiu fermentation. The fermentation properties, including weight loss of CO_2_, liquor yield, residual sugar, total acids, and total esters, were investigated to assess the stable performance of the mutants with* BAT2* deletion and* ATF1* overexpression. Weight loss was monitored during fermentation (Figure 8). H*α*5 showed the same weight loss trend with the parental strain, whereas the fermentation rate of mutant H*α*5::ATF1 was slightly slower than that of strain *α*5. However, the mutants had no significant difference in terms of total weight loss of CO_2_ compared with the parental strain (*P *> 0.05). The ethanol content, residual sugar, total acids, and total esters were detected after fermentation (Table 3). No obvious distinction was observed in the ethanol content, residual sugar, total acids, and total esters of the parental strain and H*α*5 (*P *> 0.05). Meanwhile, the content of ethanol and total acids in H*α*5::ATF1 decreased by 2.25% (16.03% v/v in parental strain and 15.67% v/v in H*α*5::ATF1,* P *< 0.05) and 19.35% (0.093 g/L total acids in parental strain, and 0.075 g/L total acids in H*α*5::ATF1,* P *< 0.05). Moreover, the total ester content increased by 111.35% (0.705 g/L in the parental strain and 1.490 g/L in H*α*5::ATF1,* P *< 0.05) compared with those of parental strain *α*5.

## 4. Discussion

*S. cerevisiae* plays a major role in traditional biotechnologies, such as baking, brewing, and wine making. Its broad application in industry is closely related to its role as a major platform for metabolic engineering that aims to enhance yeast biotechnology. Thus, the application of gene-modified technology in genome editing of* S. cerevisiae* is necessary for promoting its efficacy and safety in industry. Many current tools for gene manipulation in* S. cerevisiae* are still limited in terms of generation of the disruption construct or in the efficiency of transformation or marker removal. This study developed a rapid and highly efficient protocol for genome editing allowing gene disruption without any exogenous gene in* S. cerevisiae*. HERP1.0 cassette, including the* TkMX* marker and a galactose-inducible I-SCE1 endonuclease, was fused into a fusion DNA fragment of the upstream and the downstream sequences with an* I-SCE1* site and then transformed into the* S. cerevisiaeα*5 with an upstream sequence of target gene. The HERP1.0 cassette and the DR were inserted into the locus of the target gene after the first homologous recombination. The DSB was generated at the* I-SCE1* site under the induction of galactose and repaired through the second homologous recombination of DRs. Meanwhile, sequence analysis of the target region revealed that the HERP1.0 sequences were removed completely. The method is more effective than the two-step integration protocol described by Dong et al., in which wild-type* URA3* in host strain was replaced by a disabled* ura3* gene before deletion, and the resulting mutants of the second integration recombination of DRs were either parental or deletion strains [35]. In the current method, the* TKMX* gene that has not been identified in fungus to date was used as a selectable and counter-selectable marker, and the final mutant was only the desired deletion strain. As the mutant left only self-DNA in its native location without any foreign DNA sequence after deletion, the current strategy can be repeatedly used in yeast strains.

The desired deletion strain was obtained through the second homologous recombination after galactose induction. The length of DRs, induction time, and galactose concentration were the crucial factors that affect the frequency of the second homologous recombination efficiency. The length of DRs has a direct correlation with the DSB. In Rinji AKada's research, a 40 bp sequence derived from a region adjacent to the targeted locus was placed in an integrating construct to generate DRs after integration, and the recombination frequency (10^−6^) was low [17]. Thus, 500 bp was chosen as the optimal length of DRs to promote the second homologous recombination in the desired deletion in this study. Furthermore, the induction time and galactose concentration during galactose induction affected the expression of the I-SCE1 endonuclease and then influenced the DSB repair through homologous recombination. Considering the period and efficiency of the experiment, 24 h and 0.5 g/100 mL were chosen as the optimal induction time and galactose concentration, respectively.

Aminotransferase encoded by* BAT2* and alcohol acetyltransferases encoded by* ATF1* are related to the metabolism of branched-chain alcohols and acetate esters, respectively. The strain H*α*5 with* BAT2 *deletion and H*α*5::ATF1 with* BAT2* deletion and* ATF1* overexpression were engineered using the current method. The higher alcohol contents, specifically* n*-propanol, isobutanol, and isoamylol contents, decreased significantly after* BAT2 *gene deletion in mutant H*α*5. The result was consistent with the conclusion obtained in our previous research [36].* ATF1 *gene encoding alcohol acetyltransferases was overexpressed under the control of promoter* TEF1* at the locus of the* BAT2 *in this work. The acetate ester concentrations produce by the mutant H*α*5::ATF1 had obvious increase compared with those of the parental strain, and the higher alcohols, such as isobutanol, *β*-phenylethanol, and isoamylol production, were further decreased compared with the mutant H*α*5. This result demonstrated that* ATF1* overexpression contributed not only in the reduction of higher alcohol production but also in the improvement of the acetate ester contents. The result was in accordance with our previous study, in which the* ATF1* gene was overexpressed under the control of the promoter* PGK1 *and inserted into the* BAT2* locus with* KanMX *maker, whereas excision of* KanMX *maker left behind the foreign sequences (a single* loxP* site) [5]. In this study, the mutants were constructed with* BAT2* deletion and* ATF1* overexpression via the seamless gene deletion system, in which* TKMX* gene was used as a selectable and counter-selectable marker. No any foreign DNA sequence retained in* S. cerevisiae* chromosome after deletion, thereby increasing the security of the engineered strains in industry.

## 5. Conclusions

A rapid and highly efficient system for seamless gene deletion through endonuclease I-SCEI as a DSB inducer and two-step integration protocol was developed. To accelerate the system efficiency, the factors affecting the frequency of the second homologous recombination efficiency were screened and optimized. In addition, the strains with* BAT2 *deletion and* ATF1* overexpression were constructed through the novel method, resulting in desirable reduction of higher alcohol contents and improvement of the acetate ester production. The novel protocol proposed in this work is a promising strategy for gene deletion in* S. cerevisiae*, providing insights into further improvement of performance characteristics of* S. cerevisiae.* Moreover, as any foreign genes did not retain at chromosomes after deletion, the engineered strains can be used in industrial production in security, easing public safety concerns over genetic modification and meeting the requirement of modern science and technology and industrial production.

## Figures and Tables

**Table 1:** Strains and plasmids used in the current study.

Strains or plasmids	Relevant characteristic	Reference or source
Strains		
DH5*α*	*sup*E44 ∆*lac*U169 (*φ* 80*lac*Z∆M15) *hsd*R17 *rec*Al *end*Al *gyr*A96 *thi*1 *rel*A	This lab
yWH245	∆*ade2*::HERP1.0 ∆*leu2 *∆*ura3 *Δ*ho*::*KanMX*	Gifted by William G
*α*5	*MATα*, haploid yeast strain from AY15	This lab
H*α*5 (50)	*MATα*, Δ*bat2*::S-HERP1.0-BAT2-U_2_	This study
H*α*5 (150)	*MATα*, Δ*bat2*::S-HERP1.0-BAT2-U_3_	This study
H*α*5 (300)	*MATα*, Δ*bat2*::S-HERP1.0-BAT2-U_4_	This study
H*α*5 (500)	*MATα*, Δ*bat2*::S-HERP1.0-BAT2-U	This study
H*α*5 (700)	*MATα*, Δ*bat2*::S-HERP1.0-BAT2-U_5_	This study
H*α*5 (1000)	*MATα*, Δ*bat2*::S-HERP1.0- BAT2-U_6_	This study
H*α*5+H	*MATα*, Δ*bat2*::S-HERP1.0-BAT2-U	This study
H*α*5	*MATα*, Δ*bat2*	This study
H*α*5::ATF1+H	*MATα*, Δ*bat2*::S-HERP1.0-BA-P_TEF1_-*ATF1*-T_PGK1_	This study
H*α*5::ATF1	*MATα*, Δ*bat2*:: P_TEF1_-*ATF1*-T_PGK1_	This study
Plasmids		
pUC19	Ap^r^, cloning vector	This lab
Yep352	Ap^r^, ori control vector	This lab
pUC-BBTAP	Ap^r^, containing *BA-P*_*TEF1*_*-ATF1-T*_*PGK1*_*-BB* cassette	This lab
pUC19-UD	Ap^r^, containing BAT2-UD cassette	This study
YHERP1.0	Ap^r^, TK^r^, containing HERP1.0 cassette	This study
YHERP1.0ΔBAT2	Ap^r^, TK^r^, containing HERP1.0-BAT2-UD cassette	This study
YHERP1.0(50 bp)	Ap^r^, TK^r^, containing HERP1.0-BAT2-U_2_D cassette	This study
YHERP1.0(150 bp)	Ap^r^, TK^r^, containing HERP1.0-BAT2-U_3_D cassette	This study
YHERP1.0(300 bp)	Ap^r^, TK^r^, containing HERP1.0-BAT2-U_4_D cassette	This study
YHERP1.0(500 bp)	Ap^r^, TK^r^, containing HERP1.0-BAT2-UD cassette	This study
YHERP1.0(700 bp)	Ap^r^, TK^r^, containing HERP1.0-BAT2-U_5_D cassette	This study
YHERP1.0(1000 bp)	Ap^r^, TK^r^, containing HERP1.0-BAT2-U_6_D cassette	This study
YHERP1.0ΔBAT2::ATF1	Ap^r^, TK^r^, containing HERP1.0-BA -P_TEF1_-*ATF1*-T_PGK1_-BB cassette	This study

**Table 2:** Primers used in the present study.

Primers	Sequence (5′ → 3′)	Restriction site
BAT2-A(KpnI)-F	GG*GGTACC*CTCTACCACCTGCTGCAG	*Kpn*I
BAT2-A-R	TACGTAGCAATAGCGATACTATCGTTCTTAAAACTCGTGG	
BAT2-B-F	CCACGAGTTTTAAGAACGATAGTATCGCTATTGCTACGTA	
BAT2-B(SphI)-R	ACAT*GCATGC*TTATTTTCCGTCAATTTTCAATCTTGCGCCC	*Sph*I
YH1.0(EcoRI)-F	GACCATGATTAC*GAATTC*TGGATGGACGCAAAGAAG	*Eco*RI
Yh1.0(SphI)-R	GTGCCAAGCTT*GCATGC*GG*GGTACC*ATTAAGGGTTCTCGAGAGCT	*Sph*I
		*Kpn*I
B+50 bp-F	GG*GGTACC*AAAATTTTAGAAATTTAAGG	*Kpn*I
B+150 bp-F	GG*GGTACC*GCTGACAGTATAACTAATAT	*Kpn*I
B+300 bp-F	GG*GGTACC*CCCTCTCTGACACCTCTTGT	*Kpn*I
B+500 bp-F	GG*GGTACC*CGCTCCTTTCCAAACATCTT	*Kpn*I
B+700 bp-F	GG*GGTACC*ACAAGAACATCTCATCTACT	*Kpn*I
B+1000 bp-F	GG*GGTACC*CCACCAGTTTCACGCCTACC	*Kpn*I
BAT2-B-R	GGG*GTACCCC*TCAATCGGCACATTCATA	*Kpn*I
BAT2-A-F	GCTCCCTCCAACTACTCT	
BAT2-A-R(F)	AACTTCTTTGCGTCCATCCAAGTCGACATTACCCTGTTATCCCTAATCGTTCTTAAAACTCGTGG	I-SCEI
BAT2-B-F(R)	CCACGAGTTTTAAGAACGATTAGGGATAACAGGGTAATGTCGACTTGGATGGACGCAAAGAAGTT	I-SCEI
AHB-A-U	TTCACTGGGACCCTTTCA	
AHB-A-D	GCTTCTAATCCGTACTTCAATA	
AHB-B-U	ATGCGTCAATCGTATGTG	
AHB-B-D	TGAAATCCTTGTCCAGCT	
YH1.0ΔB::A-F	CGAGAACCCTTAAT*GGTACC*GCTCCTTTCCAAACATCTTCG	*Kpn*I
YH1.0ΔB::A-R	AAGCTTGCATGCGG*GGTACC*GCCCTCTAAAGATTCATCGG	*Kpn*I
AHB(ATF1)-A-D	GCATCGGGCTCCTCTAACTG	
AHB(ATF1)-B-U	GGGTTCAATATACAAGGCTTCG	

**Table 3:** The alcohol and residual sugar content fermented by the parental strain and engineered strains ^a^.

Strains	Alcohol content (%, v/v)	Residual sugar (g/L)	Total acids (g/L)	Total esters (g/L)
*α*5	16.03 ± 0.15	2.04 ± 0.12	0.093 ± 0.012	0.705 ± 0.009
H*α*5	16.12 ± 0.11	2.88 ± 0.10	0.100 ± 0.015	0.629 ± 0.011
H*α*5::ATF1	15.67*∗* ± 0.10	3.01 ± 0.08	0.075*∗* ± 0.010	1.490*∗* ± 0.015

^a^ Data are the average of three independent experiments ± the standard deviation. Significant difference of H*α*5::ATF1 from the parental strain was confirmed by Student's *t*-test (*∗P *< 0.05, n = 3).

## Data Availability

The data used to support the findings of this study are available from the corresponding author upon request.
